# Supplementary material for: PPTC7 maintains mitochondrial protein content by suppressing receptor-mediated mitophagy
Source: Nat Commun. 2023 Oct 13;14:6431. doi: 10.1038/s41467-023-42069-w (PMC10575892; doi:10.1038/s41467-023-42069-w)
Supplement: Supplementary file 1 — Supplementary Information [file 41467_2023_42069_MOESM1_ESM.pdf]

SUPPLEMENTARY INFORMATION

PPTC7 maintains mitochondrial protein content by suppressing receptor-mediated mitophagy

Niemi et al.

Supplementary Figure 1

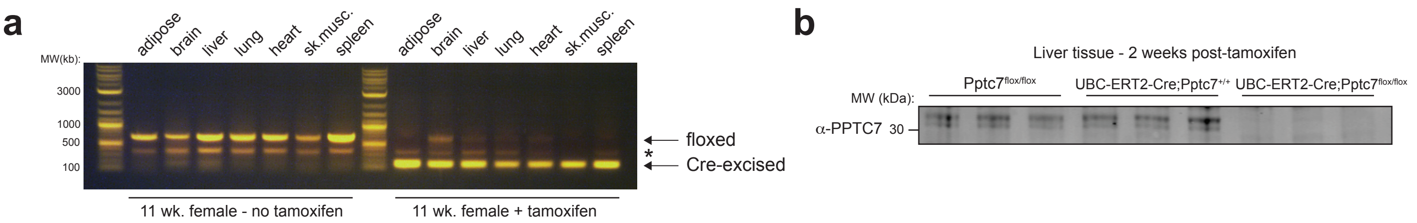

c

Significantly upregulated proteins in *Pptc7* KO liver tissue

Rank	Gene symbol	Pathway/Function (MitoCarta 3.0)
1	Pdk4	Metabolism > Carbohydrate metabolism > Pyruvate metabolism
2	Slc25a47	Small molecule transport > SLC25A family
**3**	**Bnip3**	**Mitochondrial dynamics and surveillance > Apoptosis**
4	Aifm2	Metabolism > Detoxification > ROS and glutathione metabolism | Mitochondrial dynamics and surveillance > Apoptosis
5	Abcd3	Small molecule transport > ABC transporters
**6**	**Bnip3l**	**Mitochondrial dynamics and surveillance > Apoptosis**
7	Aldh1l1	Metabolism > Vitamin metabolism > Folate and 1-C metabolism
8	Stom	MXP (no annotated function)
9	Cmpk2	Metabolism > Nucleotide metabolism > Nucleotide synthesis and processing
10	Trit1	Mitochondrial central dogma > mtRNA metabolism > mt-tRNA modifications | Mitochondrial central dogma > mtRNA metabolism > mt-rRNA modifications
11	Pxmp4	MXP (no annotated function)
12	Sqrdl	Metabolism > Electron carriers > Q-linked reactions, other | Metabolism > Sulfur metabolism
13	Cryz	MXP (no annotated function)
14	Acot11	Metabolism > Lipid metabolism > Fatty acid oxidation

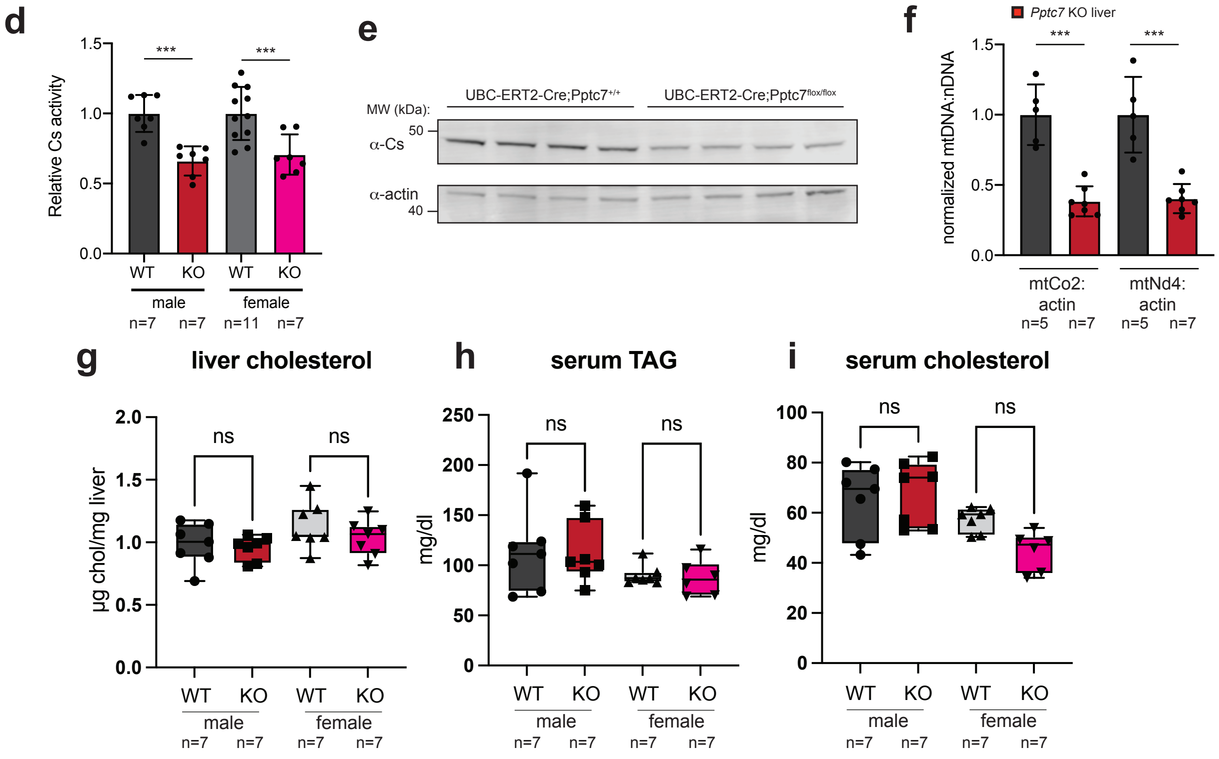

Supplementary Figure 1: *Establishment and validation of the inducible Pptc7 KO knockout mouse*. **A**. Genotyping for the floxed allele in 11-week-old female mice without (left) and with (right) one week of tamoxifen administration. Genotyping reveals substantial recombination only in the presence of tamoxifen for most tissues assayed. *represents a non-specific band. Experiment is representative of at least three independent experiments. **B**. Endogenous PPTC7 in floxed mice (left, n=3), Cre-containing wild type mice (middle, n=3), or experimental knockout animals (right, n=3). Only the experimental animals show knockout at the protein level. Experiment is representative of at least three independent experiments. **C**. Table of significantly upregulated mitochondrial proteins in *Pptc7* knockout relative to control liver tissue. BNIP3 and NIX are highlighted. **D**. Citrate synthase (Cs)) activity in liver tissue from male and female mice. Each dot represents mean Cs activity from an individual animal, n=7 for male WT, n=7 for male KO, n=11 for female WT, n=7 for female KO; error bars represent standard deviation. ***p<0.001, two-tailed Student's t test. **E**. Expression of citrate synthase protein in n=4 control (left) or n=4 experimental (right) animals. Actin is shown as a load control. Experiment is representative of at least three independent experiments. **F**. Relative mtDNA levels (compared to nuclear DNA, or nDNA) in male wild type (grey, n=5) or *Pptc7* KO (red, n=7) liver. Error bars represent standard deviation. ***p<0.001, two-tailed Student's t test. **G**. Liver cholesterol content in male and female wild-type (WT) and *Pptc7* KO mice fasted overnight. Ordinary one-way ANOVA performed. ns = not significant. The box plot extends from the 25th to 75th percentile; whiskers stretch from minimum to maximum datapoints. The line in the middle of the box plot represents the median. **H**. Serum triacylglycerol content (TAG) in male and female wild-type (WT) and *Pptc7* KO mice fasted overnight. Ordinary one-way ANOVA performed. ns = not significant. The box plot extends from the 25th to 75th percentile; whiskers stretch from minimum to maximum datapoints. The line in the middle of the box plot represents the median. **I**. Serum cholesterol content in male and female wild-type (WT) and *Pptc7* KO mice fasted overnight. Ordinary one-way ANOVA performed. ns = not significant. The box plot extends from the 25th to 75th percentile; whiskers stretch from minimum to maximum datapoints. The line in the middle of the box plot represents the median.

Supplementary Figure 2

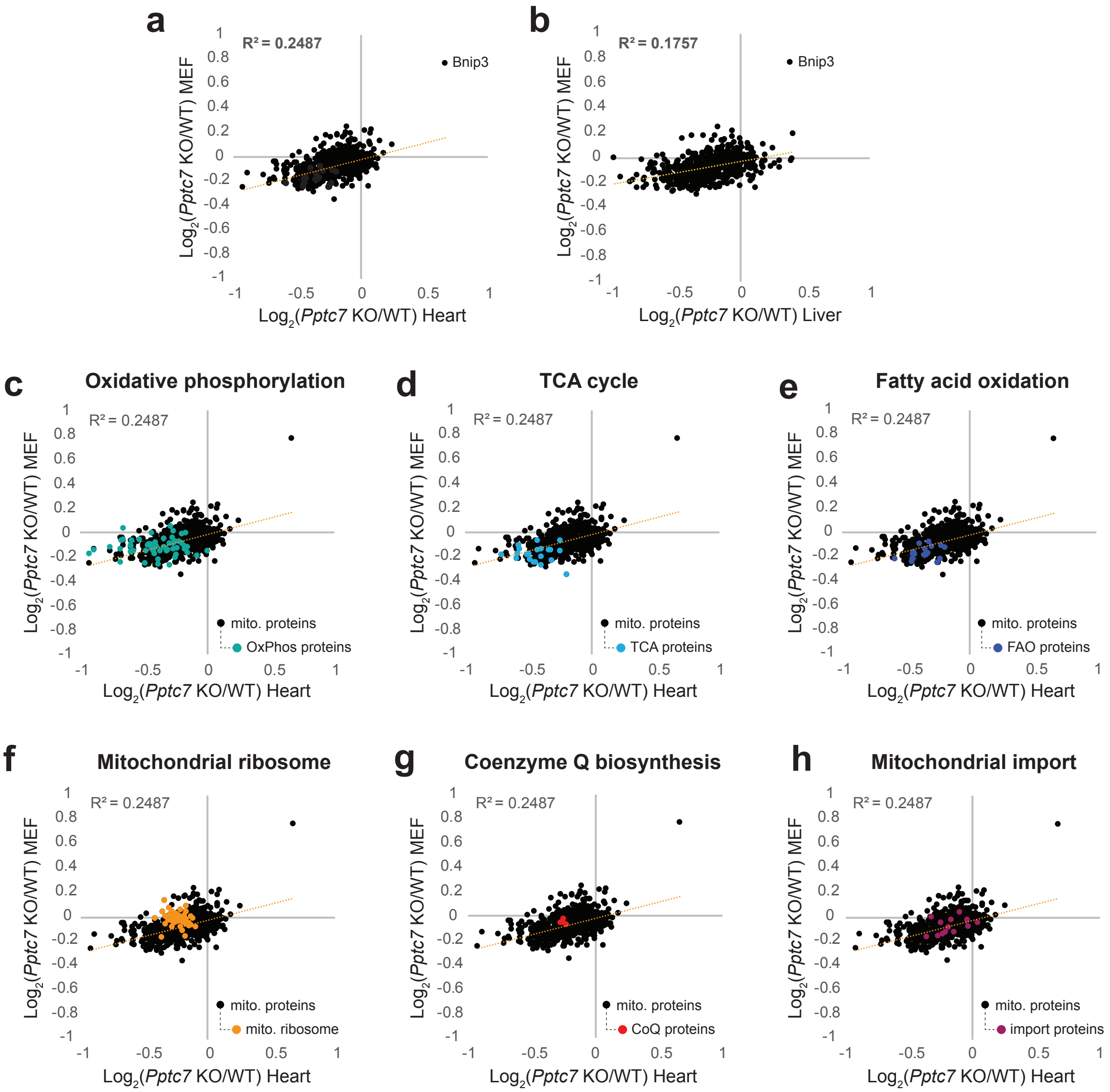

Supplementary Figure 2: *Metabolic proteins are amongst the most affected by Pptc7 KO*. **A**., **B**. Correlation analysis between mitochondrial protein fold changes in *Pptc7* KO MEFs (y-axis) and perinatal heart (x-axis, **A**.) and perinatal liver (x-axis, **B**.). **C**.-**H**., Pathway analysis highlighting proteins involved in oxidative phosphorylation (**C**.), the TCA cycle (**D**.), fatty acid oxidation (**E**.), the mitochondrial ribosome (**F**.), coenzyme Q biosynthesis (**G**.), and mitochondrial protein import (**H**.).

Supplementary Figure 3

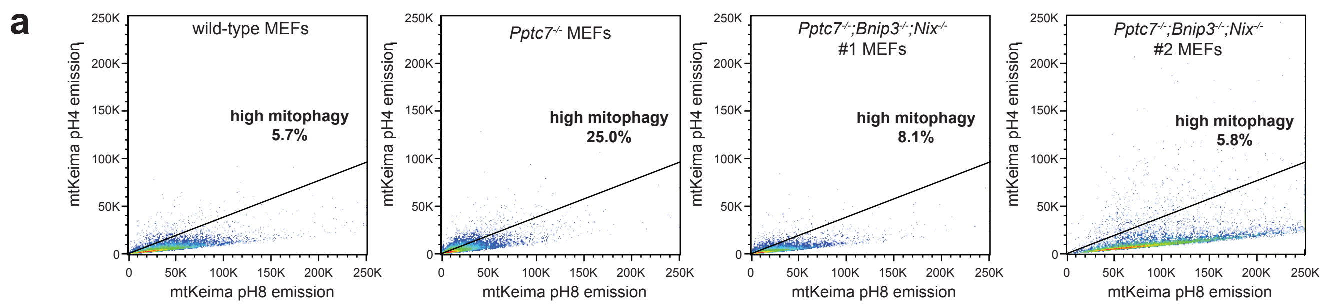

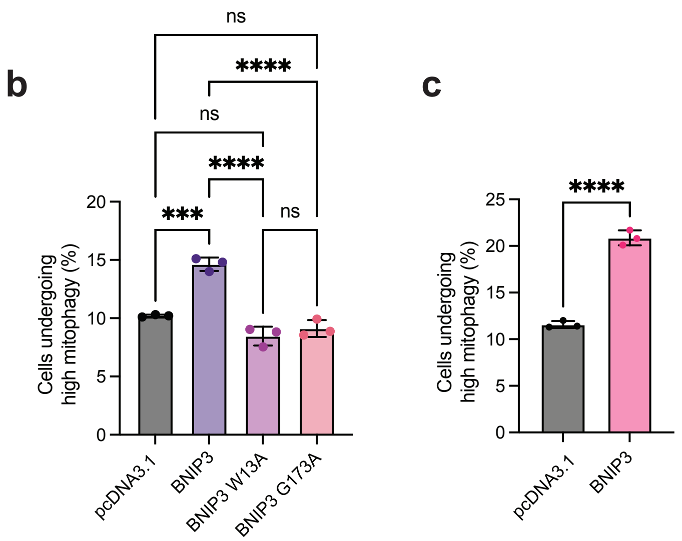

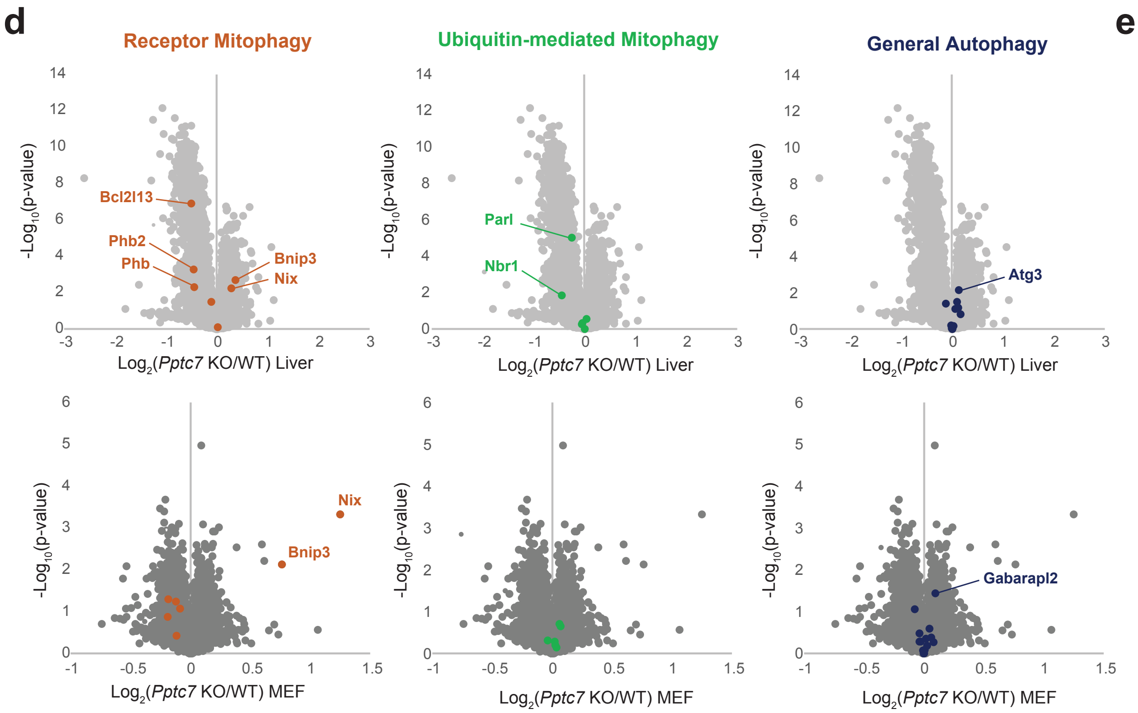

e

		MEF		Liver	
		log2FC	p value	log2FC	p value
receptor mitophagy	Bcl2l13	-0.12	0.06	-0.50	**0.00**
	Bnip3	0.76	**0.01**	0.36	**0.01**
	Bnip3l	1.25	**0.00**	0.29	**0.02**
	Fkbp8	-0.12	0.37	-0.11	0.08
	Pgam5	-0.09	0.08	0.02	0.84
	Phb	-0.19	0.13	-0.45	**0.02**
	Phb2	-0.18	0.05	-0.45	**0.00**
	Ndp52	n.q.		n.q.	
Ub-mediated mitophagy	Nbr1	0.04	0.72	-0.45	**0.04**
	Optn	0.03	0.63	-0.05	0.64
	Parl	-0.04	0.49	-0.25	**0.00**
	Sqstm1	0.07	0.22	-0.04	0.57
	Tax1bp1	0.06	0.20	0.01	0.97
	Tbk1	0.02	0.52	0.05	0.39
	Pink1	n.q.		n.q.	
	Prkn	n.q.		n.q.	
General autophagy	Atg12	-0.08	0.09	-0.12	0.10
	Atg16l1	0.02	0.43	0.00	0.99
	Atg2a	-0.03	0.52	-0.02	0.72
	Atg2b	0.01	0.82	0.10	0.08
	Atg3	0.00	0.98	0.13	**0.03**
	Atg4a	0.08	0.54		
	Atg4b	-0.04	0.52	0.00	0.98
	Atg5	-0.01	0.85	0.07	0.16
	Atg7	0.02	0.65	0.03	0.76
	Atg9a	0.05	0.25	0.01	0.90
	Becn1	0.06	0.41	0.01	0.90
	Gabarap	-0.03	0.33	-0.01	0.92
	Gabarapl1	n.q.		0.17	0.25
	Gabarapl2	0.10	**0.04**	n.q.	
	Rheb	0.00	0.99	0.13	0.14

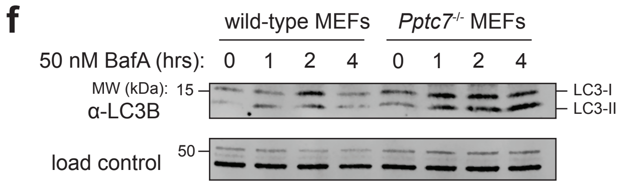

Supplementary Figure 3: *Pptc7 KO cells undergo elevated mitophagy and autophagy*. **A**. FACS histograms of mt-Keima positive wild-type, *Pptc7* KO, or *Pptc7/Bnip3/Nix* TKO cells. The diagonal line represents the gate; 'high mitophagy' cells were quantified as above this line. **B**. Quantification of cells undergoing high mitophagy rates as quantified via FACS. Wild-type MEFs were stably transfected with pcDNA3.1 (vector only), BNIP3, BNIP3 W13A (a LIR mutant) or BNIP3 G173A (a TM mutant that disrupts dimerization). Dots each represent a biological replicate; error bars represent standard deviation. Ordinary one-way ANOVA performed. **** = $p<0.0001$, *** = $p<0.001$. **C**. Quantification of cells undergoing high mitophagy rates as quantified via FACS. Wild-type MEFs were stably transfected with pcDNA3.1 (vector only) or NIX. Dots each represent a biological replicate; error bars represent standard deviation. Two-tailed Student's t-test performed. **** = $p<0.0001$, *** = $p<0.001$. **D**., **E**. Analysis of proteins identified in receptor-mediated mitophagy (orange), ubiquitination-mediated mitophagy (green), or general autophagy (navy blue) in adult liver tissue (top volcano plots) or MEFs (bottom volcano plots). Significantly changing proteins in Pptc7 KO cells or tissue relative to control cells or tissue are highlighted. **F**. Table of all quantified proteins involved in receptor-mediated mitophagy (orange), ubiquitination-mediated mitophagy (green), or general autophagy (navy blue). Experiment is representative of at least three independent experiments. G. Wild-type or *Pptc7* KO MEFs were treated with 50 nM bafilomycin A for the indicated times. Cells were lysed, run on SDS-PAGE, and Western blotted for LC3. Source data included in Source data file.

Supplementary Figure 4

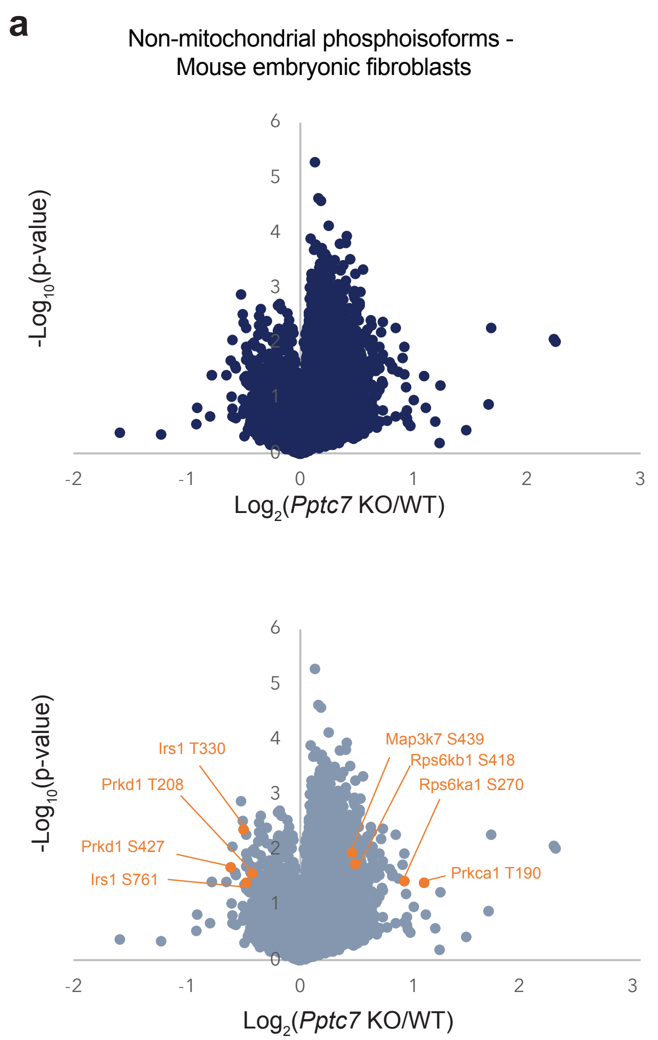

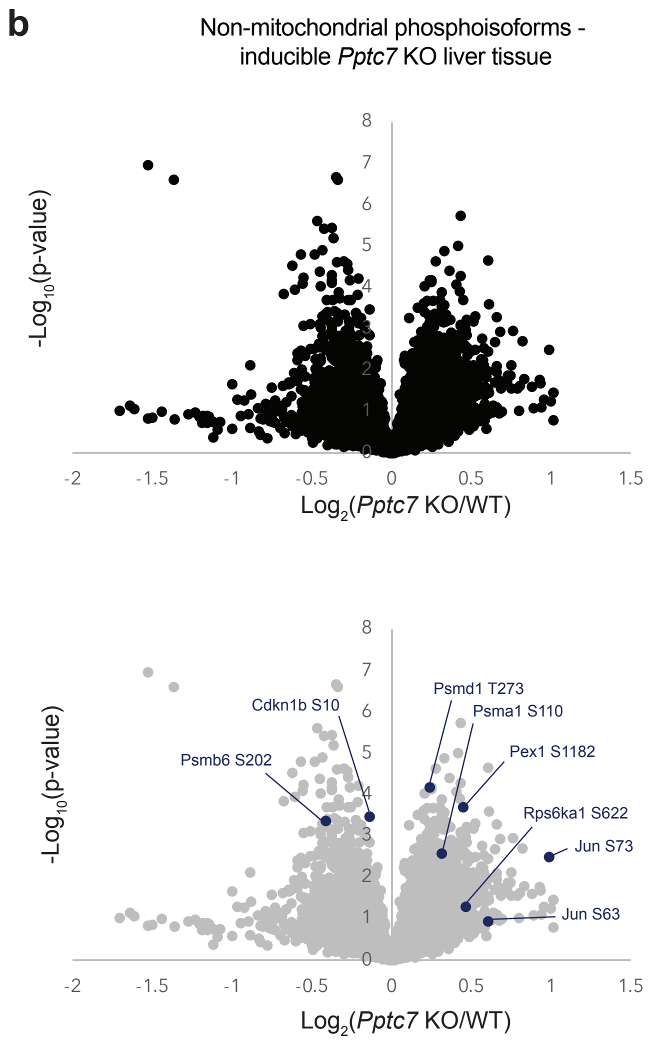

Supplementary Figure 4: *Non-mitochondrial phosphoproteome analysis from Pptc7 knockout cells and tissues.* **A**., B. Non-mitochondrial phosphopeptides shown as individual dots as quantified in MEFs (left) or adult mouse liver tissue (right). Distributions of total phosphoproteomes on top; highlights of select non-mitochondrial phosphorylation sites of interest on bottom.

Supplementary Table 1

Niemi et al. *Pptc7 maintains mitochondrial protein content by suppressing receptor-mediated mitophagy*

Sequences of CRISPR reagents used to generate the *Pptc7* conditional mouse (Int23 and Int24) and the sequence of the resulting floxed allele found in the *Pptc7* conditional mouse model are found below.

**CRISPR reagents**	
Int23	5’-CTGGCACTCGTGTCTACAGG_AGG-3’
Int34	5-‘TGCAAGGGACTTGGAATAAG_TGG-‘3
**Resulting floxed *Pptc7* allele sequence**	
TTTGGAGGCTGGGTATGTCCCTGTCTGAAACATCAGTAAATTTAGTGAAGTGTCAGCTGC<br>AGGAACCTTGTTCCAACGACTAAGTAGTAAGACCCAGTAACCTGCTGTGGGGCCTGGCA<br>CTCGTGTCTACATAACTTCGTATAATGTATGCTATACGAAGTTATAGGAGGAGGAACATG<br>GTGCACCTTGCTTACCGTGCTCCCACACCCCTTGTCCTCAGCACCTCGGGGTTCTCGGG<br>TGTGGGGCAAACCTGGATGGAGCTGAGTGCTGACGGGCAGCAGCCCGGGGGAGACAG<br>TGAGCGGATGCCCATGTTGTTTGTAGGTAGCAGCACAGCCTGCATCGTGGTGCTGGACA<br>GAAGTAGCCACCGCTTGCACACAGCGAACCTGGGTGACTCGGGCTTCCTGGTGGTCCG<br>GGGCGGCGAGGTTGTGCACCGGTCTGACGAGCAGCAGCACTACTTCAACACTCCATTC<br>CAGCTCTCCATCGCCCCTCCTGAGGCCGAGGGGGTTGTCCTGAGCGACAGGTAAGCAA<br>GCAGGAGGTGCCTCACCCATCCCTCCCTTCCTCCCTCCTGTCCTCTTTCCTGAGACACT<br>GACGTCCTCAGGACCAGTTAGTGGGGCCTTTGCAAGGGACTTGGAAATAACTTCGTATA<br>ATGTATGCTATACGAAGTTATTAAGTGGGTATCCCAGCCAAGCCAAGGTGCTCTTACAGA<br>GTCAGCTGGAAGTGTTGTGCACAGGCCAGTCCGCCTTCTGTCAGTCCAGTCCTCGGGC<br>AGCCGCACACTGGCTGACGGCCGTCTTACTTGTCAGAATAACTTCCCACTCAGAGGTCC<br>TTCTCACACATGCGCCCAACAGAAGAGAAGTGGCCAGACCTGCCTCCGTCCTTAATGGG<br>GTCACTAGGGTGGTCTCTTTTTCTTGATGATTGCTAATGTCTTAGTTTAATTTTAAACTTCC<br>AGGAACCTTTCAAAACAACTG<br><br>LoxP sites in red<br>Exon 3 in blue	

Supplementary Table 2

Niemi et al. *Pptc7 maintains mitochondrial protein content by suppressing receptor-mediated mitophagy*

Sequences of primers used to genotype the *Pptc7* conditional mouse. Sequences amplify wild type or floxed allele (as full length or excised/knockout) and the UBC-Cre-ER^{T2} recombinase used in this study.

**Primer name**	**Sequence**
Pptc7 flox forward	5'-TAGTGAAGTGTCAGCTGCAGGAA-3'
Pptc7 flox reverse	5'-GACTCTGTAAGAGCACCTTGGC-3'
Cre forward	5'-GACGTCACCCGTTCTGTTG-3'
Cre reverse	5'-AGGCAAATTTTGGTGTACGG-3'

Supplementary Table 3

Niemi et al. *Pptc7 maintains mitochondrial protein content by suppressing receptor-mediated mitophagy*

Sequences of primers used to amplify nuclear DNA (β-actin) and mitochondrial DNA (mtDNA, Mt-Co2 and Mt-Nd4).

**Primer name**	**Sequence**
β-actin fwd	5’-GGCTGTATTCCCCTCCATCG-3’
β-actin rev	5’-CCAGTTGGTAACGCCATGT-3’
Mt-Co2 fwd	5’-CGAGTCGTTCTGCCAATAGAA-3’
Mt-Co2 rev	5’-CCTGGTCGGTTTGATGTTACT-3’
Mt-Nd4 fwd	5’-GCCTCACATCATCACTCCTATT-3’
Mt-Nd4 rev	5’-GGCTATAAGTGGGAAGACCATT-3’

Supplementary Table 4
Niemi et al. *Pptc7 maintains mitochondrial protein content by suppressing receptor-mediated mitophagy*

Sequences guide RNAs (gRNAs) and primers for detecting *Bnip3* and *Bnip3l* deletion products post-CRISPR.

**gRNA name**	**gRNA sequence**
m.Bnip3.5'.sp2	5’-TCCTGGGAATGTGAATGGGC-3’
m.Bnip3.5'.sp6	5’-AAGTCATCAAGCCCCACAAG-3’
m.Bnip3.3'.sp17	5’-ATCTGGGGCTCAAGTCTAGA-3’
m.Bnip3.3'.sp9	5’-CCAGGTCTGAGGCCACATTT-3’
m.Bnip3l.5'.sp10	5’-TATTATCTGACTCGTCCAAC-3’
m.Bnip3l.5'.sp4	5’-TTATCTGAACTCAAGCATGT-3’
m.Bnip3l.3'.sp1	5’-TGTAGCGTACATGTTTAAAC-3’
m.Bnip3l.3'.sp14	5’-TAAGCACTTGATGTCAAACT-3’
**Primers for deletion and inside PCR**	
Bnip3 deletion – m.Bnip3.5F	5’-TGGCCTCTGGTAAATCACAGA-3’
Bnip3 deletion – m.Bnip3 3R	5’-GTAGTGCTGCCTAGGAACCG-3’
Bnip3 inside – m.Bnip3.inside.F	5’-TAGTCCTGCCTAGCCTTGAA-3’
Bnip3 inside – m.Bnip3.inside.R	5’-GCAACCCACCCACAAAAACAT-3’
Bnip3l deletion – m.Bnip3.5F	5’-AAACTAACTGGCTGGAGAGC-3’
Bnip3l deletion – m.Bnip3.3R	5’-GTGTATATGTGGAAGCCAGAGGA-3’
Bnip3l inside – m.Bnip3l.inside.F	5’-GTTCCTTCCTCGTCTTCCATCCA-3’
Bnip3l inside – m.Bnip3l.inside.R	5’-GCAAATGAGCTTGCCCCTTGTGA-3’
