# Supplementary material for: PPTC7 maintains mitochondrial protein content by suppressing receptor-mediated mitophagy
Source: Nat Commun. 2023 Oct 13;14:6431. doi: 10.1038/s41467-023-42069-w (PMC10575892; doi:10.1038/s41467-023-42069-w)
Supplement: Supplementary file 3 — Description of Additional Supplementary Files [file 41467_2023_42069_MOESM3_ESM.pdf]

Description of Additional Supplementary Files

File name: Supplementary Data 1

Description: Inducible Liver proteomics

File name: Supplementary Data 2

Description: MEF proteomics

File name: Supplementary Data 3

Description: TKO MEF proteomics
