# Supplementary material for: PPTC7 maintains mitochondrial protein content by suppressing receptor-mediated mitophagy
Source: Nat Commun. 2023 Oct 13;14:6431. doi: 10.1038/s41467-023-42069-w (PMC10575892; doi:10.1038/s41467-023-42069-w)
Supplement: Supplementary file 7 — Reporting Summary [file 41467_2023_42069_MOESM7_ESM.pdf]

nature portfolio

Corresponding author(s): Natalie M. Niemi and David J. Pagliairini

Last updated by author(s): Sep 20, 2023

Reporting Summary

Nature Portfolio wishes to improve the reproducibility of the work that we publish. This form provides structure for consistency and transparency in reporting. For further information on Nature Portfolio policies, see our Editorial Policies and the Editorial Policy Checklist.

Statistics

For all statistical analyses, confirm that the following items are present in the figure legend, table legend, main text, or Methods section.

n/a	Confirmed	
☐	☒	The exact sample size (*n*) for each experimental group/condition, given as a discrete number and unit of measurement
☐	☒	A statement on whether measurements were taken from distinct samples or whether the same sample was measured repeatedly
☐	☒	The statistical test(s) used AND whether they are one- or two-sided<br>*Only common tests should be described solely by name; describe more complex techniques in the Methods section.*
☐	☒	A description of all covariates tested
☐	☒	A description of any assumptions or corrections, such as tests of normality and adjustment for multiple comparisons
☐	☒	A full description of the statistical parameters including central tendency (e.g. means) or other basic estimates (e.g. regression coefficient) AND variation (e.g. standard deviation) or associated estimates of uncertainty (e.g. confidence intervals)
☐	☒	For null hypothesis testing, the test statistic (e.g. *F*, *t*, *r*) with confidence intervals, effect sizes, degrees of freedom and *P* value noted<br>*Give P values as exact values whenever suitable.*
☒	☐	For Bayesian analysis, information on the choice of priors and Markov chain Monte Carlo settings
☒	☐	For hierarchical and complex designs, identification of the appropriate level for tests and full reporting of outcomes
☒	☐	Estimates of effect sizes (e.g. Cohen's *d*, Pearson's *r*), indicating how they were calculated

Our web collection on statistics for biologists contains articles on many of the points above.

Software and code

Policy information about availability of computer code

Data collection	Western blots and genotyping gels were imaged on a LiCOR Odyssey using Image Studio software (v.5.2). Proteomics and phosphoproteomics weredatasets were collected on an Orbitrap Eclipse using a Nanospray Flex ionization source (Thermo). BioLog data was collected on an OmniLog instrument. Citrate synthase activity data was collected using a Cytation 3 plate reader using Gen5 software (BioTek, version 3.5). mtDNA analysis was performed on aQuantStudio 6 Real-Time PCR system using QuantStudio Real-Time PCR v1.2 software. mt-Keima imaging was acquired using a Leica DMi8 SP8 Inverted Confocal microscope. mt-Keima flow cytometry was performed on a LSR-Fortessa (BD Biosciences) flow cytometer using BDFACSDiva software (version 9.0).
Data analysis	Western blots were analyzed with Image Studio software. Thermo RAW files were processed with MaxQuant (version 1.5.2.8)2, implementing the Andromeda3 algorithm to perform database searching of MS2 spectra against a database of canonical proteins and isoforms (Uniprot, Mus musculus, 2018). Seahorse analysis was performed using Seahorse XF software and Prism (version 10). BioLog data was analyzed using OmniLog software and Prism (version 10). Citrate synthase activity data was analyzed using Gen5 software (BioTek, version 3.5) and Prism (version 10). Statistical analysis for biological data was performed in Prism (version 10). mtDNA analysis using QuantStudio Real-Time PCR v1.2 software and Prism (version 10). mt-Keima imaging was quantified with Image J/FIJI software, and subsequent data were analyzed in Prism (version 10). mt-Keima FACS data were quantified using Flo-Jo (version 10.8.2) and subsequently analyzed in Prism (version 10).

For manuscripts utilizing custom algorithms or software that are central to the research but not yet described in published literature, software must be made available to editors and reviewers. We strongly encourage code deposition in a community repository (e.g. GitHub). See the Nature Portfolio guidelines for submitting code & software for further information.

Data

Policy information about availability of data

All manuscripts must include a data availability statement. This statement should provide the following information, where applicable:

- Accession codes, unique identifiers, or web links for publicly available datasets
- A description of any restrictions on data availability
- For clinical datasets or third party data, please ensure that the statement adheres to our policy

The raw data for the proteomics and phosphoproteomics datasets in this study have been deposited to the MassIVE database under the accession number MSV000091194 [https://massive.ucsd.edu/ProteoSAFe/dataset.jsp?task=01739d9c0ef34ceda1a63cf4bbe56bb0]. Source data are provided with this paper.

Research involving human participants, their data, or biological material

Policy information about studies with human participants or human data. See also policy information about sex, gender (identity/presentation), and sexual orientation and race, ethnicity and racism.

Reporting on sex and gender	n/a
Reporting on race, ethnicity, or other socially relevant groupings	n/a
Population characteristics	n/a
Recruitment	n/a
Ethics oversight	n/a

Note that full information on the approval of the study protocol must also be provided in the manuscript.

Field-specific reporting

Please select the one below that is the best fit for your research. If you are not sure, read the appropriate sections before making your selection.

☒ Life sciences ☐ Behavioural & social sciences ☐ Ecological, evolutionary & environmental sciences

For a reference copy of the document with all sections, see nature.com/documents/nr-reporting-summary-flat.pdf

Life sciences study design

All studies must disclose on these points even when the disclosure is negative.

Sample size	Samples sizes for mouse studies were chosen based on power calculations based on previously determined standard deviations in associated assays. For some methods (e.g., TMT proteomics), limitations in the number of samples that could be analyzed at once influenced final number of samples analyzed. For other assays (e.g., Seahorse), the number of wells analyzed reflected how many replicates would fit on one plate for analysis.
Data exclusions	No data were excluded from analyses.
Replication	Multiple biological replicates were generated across our study. For mouse studies, at least 4 replicates were used for each study across each genotype of interest and across sexes. For cell work in the fibroblast model, six independent embryos were used to generate three wild-type and three Pptc7 knockout cell lines (Figure 2). For the BNIP3/NIX work, two independent triple knockout lines were generated and characterized. All attempts at replication of results were successful.
Randomization	For mouse studies, mice were randomly designated an identification number that was used throughout the study, which was used for identification independent of mouse genotype. Mice were randomly assigned to experimental groups based on sex and genotype. Litters usually contained matched pairs of control and experimental mice.
Blinding	For mouse studies, mice were randomly pre-assigned to cohorts and identified only by number. In general, mouse samples were processed in numerical order and mice were sorted by sex and genotype after data collection. For other studies, blinding was not necessary or achievable due to the need to allocate appropriate numbers of samples across experimental conditions, or for analysis purposes (e.g., appropriate order of running samples on gels).

Reporting for specific materials, systems and methods

We require information from authors about some types of materials, experimental systems and methods used in many studies. Here, indicate whether each material, system or method listed is relevant to your study. If you are not sure if a list item applies to your research, read the appropriate section before selecting a response.

Materials & experimental systems

n/a	Involved in the study
	☒ Antibodies
	☒ Eukaryotic cell lines
☒	☐ Palaeontology and archaeology
	☒ Animals and other organisms
☒	☐ Clinical data
☒	☐ Dual use research of concern
☒	☐ Plants

Methods

n/a	Involved in the study
☒	☐ ChIP-seq
	☒ Flow cytometry
☒	☐ MRI-based neuroimaging

Antibodies

Antibodies used	anti-Pptc7 (Novus Biologicals, catalog #NBP1-90654, 1:1000 dilution, 48 hour incubation)<br>anti-Citrate synthase (Cell Signaling Technologies, catalog #14309, 1:1000 dilution, overnight incubation)<br>anti-beta actin (Abcam, catalog # ab170325, 1:1000 dilution, overnight incubation)<br>anti-Bnip3 (rodent specific antibody, Cell Signaling Technologies, catalog #3769, 1:1000 dilution, 48 hour incubation)<br>anti-Nix (Cell Signaling Technologies, catalog #12396, 1:1000 dilution, overnight incubation)<br>anti-LC3B (Cell Signaling Technologies, catalog #3868, 1:1000 dilution, overnight incubation)<br>anti-BNIP3 (Abcam, clone EPR4034, catalog #ab109362, WB 1:1000)<br>anti-FLAG (Sigma-Aldrich, catalog #SAB4301135, WB 1:1000)<br>anti-vinculin (SCBT, clone G-11; sc-55465, WB 1:1000)
Validation	The Pptc7 antibody was validated using Pptc7 KO cells in this paper (Figure 2B).<br>The Bnip3 and Nix antibodies were validated using Pptc7/Bnip3/Nix triple KO cells in this paper (Figure 3A).<br>All other antibodies were validated by the manufacturers and were selected due to their detection of endogenous proteins near or at the expected molecular weights.

Eukaryotic cell lines

Policy information about cell lines and Sex and Gender in Research

Cell line source(s)	Mouse embryonic fibroblasts were generated from a cross of Pptc7+/- heterozygous mice, generating both wild-type (WT) and Pptc7 KO (KO) lines. Each of the TKO cell lines used in this study were generated from a Pptc7 KO clone using CRISPR. The sex of the cells was not determined as male/female embryos are morphologically indistinguishable at E14.5.
Authentication	Cells were not authenticated as they were generated in our laboratory. Cells were validated at the genomic (Figure 2A) and protein (Figure 2B) levels.
Mycoplasma contamination	Cells routinely tested negative for mycoplasma every 6 months.
Commonly misidentified lines<br>(See ICLAC register)	No commonly misidentified lines were used in this study.

Animals and other research organisms

Policy information about studies involving animals; ARRIVE guidelines recommended for reporting animal research, and Sex and Gender in Research

Laboratory animals	Pptc7 floxed animals were generated at the Biotechnology Center at the University of Wisconsin-Madison on a C57BL/6J background. Pptc7 floxed animals were bred with UBC-Cre-ERT2 transgenic mice (B6.Cg-NdorlTg(UBC-cre/ERT2)1Ejb/1J, Strain #007001, Jackson labs). All animals were 11-12 weeks old at the end of this study. Mice were housed on a 12-h light:dark cycle, and group housed by strain and sex under temperature- and humidity-controlled conditions and received ad libitum access to water and food.
Wild animals	No wild animals were used in this study.
Reporting on sex	Most studies in this manuscript use both male and female mice. In the supplemental figures, genotyping was performed on tamoxifen-treated versus non-tamoxifen treated female mice, as we predicted that tamoxifen 'leakiness' would be more of a concern in females due to their higher levels of estrogen.
Field-collected samples	No field collected samples were used in this study.
Ethics oversight	All animal work complied with ethical regulations for animal testing and research, and was done in accordance with IACUC approval by the College of Agricultural and Life Sciences (CALS) Animal Care and Use Committee at the University of Wisconsin-

Madison (protocol/animal welfare assurance #A3368-01) or at Washington University School of Medicine (animal welfare assurance D1600245.)

Note that full information on the approval of the study protocol must also be provided in the manuscript.

Flow Cytometry

Plots

Confirm that:

☒ The axis labels state the marker and fluorochrome used (e.g. CD4-FITC).

☒ The axis scales are clearly visible. Include numbers along axes only for bottom left plot of group (a 'group' is an analysis of identical markers).

☒ All plots are contour plots with outliers or pseudocolor plots.

☒ A numerical value for number of cells or percentage (with statistics) is provided.

Methodology

Sample preparation	Cells were harvested by trypsinization and were resuspended in FluoroBrite media with 0.8% heat-inactivated FBS immediately prior to flow cytometry.
Instrument	Cells were analyzed on a LSR-Fortessa (BD Biosciences; Franklin Lakes, NJ, USA) flow cytometer.
Software	Cells were analyzed using BDFACSDiva software (version 9.0).
Cell population abundance	Cells were collected to a final number of 10,000 cells per condition post-sort and post-gating strategies.
Gating strategy	Cells were gated to select for live cells, single cells, and mtKeima positive cells sequentially.

☒ Tick this box to confirm that a figure exemplifying the gating strategy is provided in the Supplementary Information.
